# Biostimulation of Bacteria in Liquid Culture for Identification of New Antimicrobial Compounds

**DOI:** 10.3390/ph14121232

**Published:** 2021-11-27

**Authors:** Hooman Mirzaee, Emily Ariens, Mark A. T. Blaskovich, Richard J. Clark, Peer M. Schenk

**Affiliations:** 1 Plant-Microbe Interactions Laboratory, School of Agriculture and Food Sciences, The University of Queensland, Brisbane, QLD 4072, Australia; e.ariens@uq.edu.au; 2 Centre for Superbug Solutions, Institute for Molecular Bioscience, The University of Queensland, Brisbane, QLD 4072, Australia; m.blaskovich@imb.uq.edu.au; 3 Peptide Chemical Biology Laboratory, School of Biomedical Sciences, The University of Queensland, Brisbane, QLD 4072, Australia; richard.clark@uq.edu.au

**Keywords:** antibiotic, antimicrobial compound, antimicrobial peptide, food pathogen, plant pathogen

## Abstract

We hypothesized that environmental microbiomes contain a wide range of bacteria that produce yet uncharacterized antimicrobial compounds (AMCs) that can potentially be used to control pathogens. Over 600 bacterial strains were isolated from soil and food compost samples, and 68 biocontrol bacteria with antimicrobial activity were chosen for further studies based on inhibition assays against a wide range of food and plant pathogens. For further characterization of the bioactive compounds, a new method was established that used living pathogens in a liquid culture to stimulate bacteria to produce high amounts of AMCs in bacterial supernatants. A peptide gel electrophoresis microbial inhibition assay was used to concurrently achieve size separation of the antimicrobial peptides. Fifteen potential bioactive peptides were then further characterized by tandem MS, revealing cold-shock proteins and 50S ribosomal proteins. To identify non-peptidic AMCs, bacterial supernatants were analyzed by HPLC followed by GC/MS. Among the 14 identified bioactive compounds, 3-isobutylhexahydropyrrolo[1,2-a]pyrazine-1,4-dione and 2-acetyl-3-methyl-octahydropyrrolo[1,2-a]piperazine-1,4-dione were identified as new AMCs. Our work suggests that antimicrobial compound production in microbes is enhanced when faced with a threat from other microorganisms, and that this approach can rapidly lead to the development of new antimicrobials with the potential for upscaling.

## 1. Introduction

Increasing antimicrobial resistance (AMR) in human, food, and plant pathogens severely limits our ability to treat diseases, and new antibiotics must be developed [1,2,3,4]. To face this challenge, new sources, approaches and strategies are essential for discovering new antimicrobial compounds. These may come from sources which have not been well investigated; for example, from unculturable or hard to culture microbes from microbiomes where the microbes need to compete heavily against each other. Furthermore, looking for less traditional antimicrobial compounds (AMCs) that may have been overlooked in previous natural product searches, such as antimicrobial peptides (AMPs), could be a potential solution to overcome AMR threats. Based on their overwhelming biodiversity, microbial communities from different environmental sources, such as soils, are a promising source for identifying new AMCs [5,6].

Bacteria, including bacteria that could be used as biocontrol agents, produce a wide range of AMCs, including AMPs and antimicrobial metabolites (AMMs) [7]. They can be found in diverse environments, including the soil, water, food spoilage and animal guts. These microbiomes harbor different culturable and non-culturable potential biocontrol bacteria that have the capacity to be used as either directly as biopesticides or indirectly to produce stable and scalable AMCs if we find a way to harness them. Appropriate screening procedures (culture-dependent or culture-independent approaches) for selecting these novel biocontrol agents is the first essential step to suppress “superbugs” [7,8,9]. Furthermore, improvements in bacterial AMP and AMM production are required, as these currently are at relatively low levels and usually occur during the stationary phase of the bacterial life cycle, which requires more costly batch cultivation rather than continuous cultivation [10,11].

In the present study, we set up a new discovery platform for AMCs produced by bacteria from soil and food spoilage samples. It has been suggested that interspecific bacterial interactions play a key role in antibiotic production [12,13], but this approach has not yet been developed for liquid cultures that can be used for larger-scale AMC production. For these reasons, we have hypothesized that the direct application of living plant pathogens (phytopathogens) can also activate defensive pathways in potential biocontrol bacteria (antagonizing bacteria) and enhance their AMC production, revealing potential AMPs and AMMs.

## 2. Results

### 2.1. Culture-Dependent Plate Assay and Identification of Bacterial Isolates

From more than 600 different single bacterial colonies that were isolated from a soil sample, 60 isolates were found to possess antimicrobial activity against phytopathogens (Gram-negative *Pseudomonas syringae* pv. *tomato* DC3000 and Gram-positive *Clavibacter michiganensis* subsp. *michiganensis*) and two food pathogens (Gram-negative *Escherichia coli* and Gram-positive *Listeria monocytogenes*) (Supplementary File S1, Figure S1 and Table S1). Interestingly, most of the isolates coming from the soil were often outcompeted when they were incubated with food pathogens. This was likely due to the bacterial isolation conditions, which had 28 °C as the basic temperature for the initial isolation of environmental samples rather than 37 °C, which was the temperature required for the food pathogen inhibition assays. In addition, six isolates from food spoilage samples (1M, 23MR, 25MR, 12LGF, 14LGF and 44LGF) showed antimicrobial activity against various pathogens, while other food spoilage isolates did not show such zones of inhibition.

After screening against pathogens, 16S rDNA amplicons for each biocontrol isolate were sequenced. Although 16S rDNA sequence BLAST searches could not fully identify the actual species, most of the bacteria came from the orders of Bacillales, Coccus, Enterobacteriales and Rhizobiales (Supplementary File S1, Table S2). Most bacteria discovered in this study belonged to the Bacillales order. *Enterobacter ludwigii* and *Enterobacter* *cloacae*, *Proteus vulgaris* and *Klebsiella pneumoniae* were placed in the Enterobacteriales. *Ochrobactrum grignonense*/*pseudogrignonense* and *Pseudochrobactrum kiredjianiae* were classified as Rhizobiales. In addition, *Comamonas jiangduensis*, *Microbacterium oxydans* and *Corymbia flavescens* belong to the Actinomycetales, Burkholderiales and Corynebacteriales, respectively.

### 2.2. Development of an Antimicrobial Discovery Pipeline

**Figure 1:**
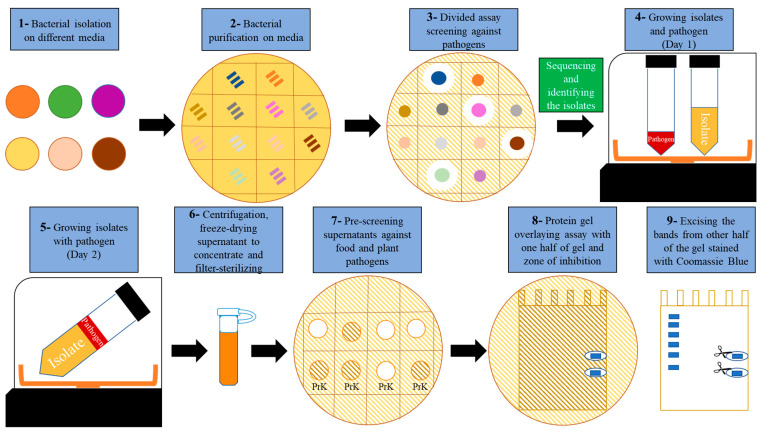
AMC antimicrobial discovery platform. Steps include (1) the isolation of microbes from environmental samples on different media (Supplementary File S1, Table S3), (2) purifying and (3) screening by plate growth inhibition assays using plant and food pathogens (hatched background), (4, 5) the AMC induction method by co-cultivation with living Gram-positive and -negative pathogens at 28 °C, (6) centrifugation and concentration of cell-free supernatants by freeze-drying, (7) testing of the concentrated supernatants with or without proteinase K to distinguish between AMPs and AMMs, followed by (8) peptide gel microbial growth inhibition assays to (9) extract bioactive peptides and MS/MS of trypsin-digested peptides or coculture supernatant fractionation by HPLC (high-performance liquid chromatography) followed by MS/MS (tandem mass spectrometry) or GC/MS (gas chromatography–mass spectrometry) analysis.

To effectively screen soil and food spoilage microbes for novel AMCs, a multistep biodiscovery pipeline was established (Figure 1). It includes the stimulation of AMC production by exposure to living plant pathogens (*Clavibacter michiganensis* subsp. *michiganensis* (*Cmm*) and *Pseudomonas syringae* pv. *tomato* DC3000 (*Pst*)), followed by plate and peptide gel microbial growth inhibition assays combined with MS/MS (Figure 1). Furthermore, the coculture supernatants (Figure 1 Step 6) could be fractionated by HPLC and analyzed by MS/MS and GC/MS, respectively.

### 2.3. Stimulation of AMC Production by Exposure to Living Plant Pathogens

Bacteria are opportunistic organisms and we hypothesized that AMCs may only be produced in large quantities when they are needed for bacteria to survive or compete. For this reason, all of the 68 antagonistic isolates that we identified based on exhibiting antimicrobial activity against the phytopathogens *Pst* or *Cmm* on agar (Figure 1 Step 3) were then co-cultured with the same phytopathogens in broth (Figure 1, Steps 4 and 5). We chose these Gram-positive and Gram-negative plant pathogens as safe pathogens (for humans) to induce antimicrobial production in potential biocontrol bacteria. To our knowledge, the induction of AMC biosynthesis by exposure to phytopathogens in a liquid culture is a novel method that has been developed in our laboratory [12,13]. As phytopathogens evolved separately from animal pathogens, most of them are safe in the case of exposure during the production phases [14]. However, the optimization may differ, depending on the bacterial isolate, the type of pathogen, the concentration, the volume and other environmental factors used to induce AMCs production in liquid culture. To effectively assay for the production of potential AMCs, 16 mL of supernatant was concentrated to ~1.5 mL using a freeze-dryer (Figure 1 Step 6), and 10 µL was subjected to plate inhibition assays with different plant and food pathogens as bacterial lawns (Supplementary File S1, Figure S2). Out of 68 isolates tested, 26 were found to produce AMCs that were found in the medium following the induction method. These were then assayed for the presence of AMPs: AMP activity could be attributed to 22 isolates (Figure 1, Step 7; Figure S2), as their activity was impaired after treatment with proteinase K (a serine protease that is used to digest proteins and peptides). Nine isolates were assumed to produce AMMs, since their activity remained unaffected by proteinase K digestion when tested against distinct pathogens (Table 1). Five isolates producing AMPs were induced by *Pst* and the rest were induced by *Cmm*. Interestingly, four isolates possessed AMP activity against one pathogen and AMM activity against another pathogen. Forty-two isolates out of 68 isolates did not show antimicrobial activity in this broth induction assay. These bacterial isolates were retested, and we found that neutralizing the pH to 7 after co-cultivation gave rise to 10 more isolates that showed antimicrobial activity. It should also be noted that the stimulation of AMC production by living plant pathogens was dose-dependent in 19YE, as with a higher dose (~4× higher OD) of phytopathogens, no antimicrobial activity was observed in this isolate.

### 2.4. Peptide Gel Microbial Inhibition Assay and Partial Identification of Antimicrobial Peptides

**Figure 2:**
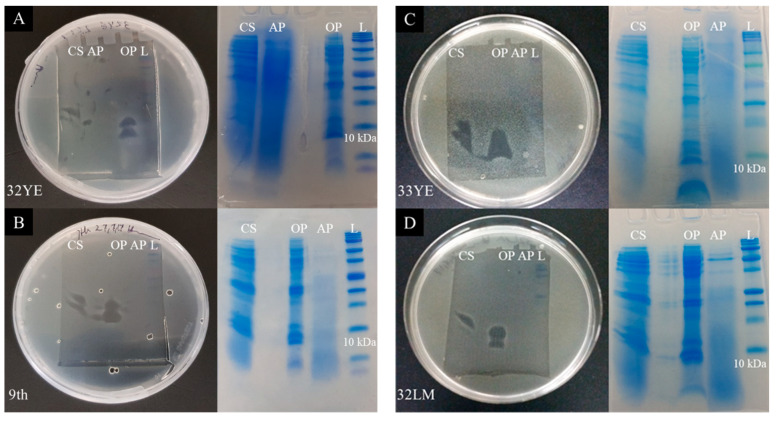

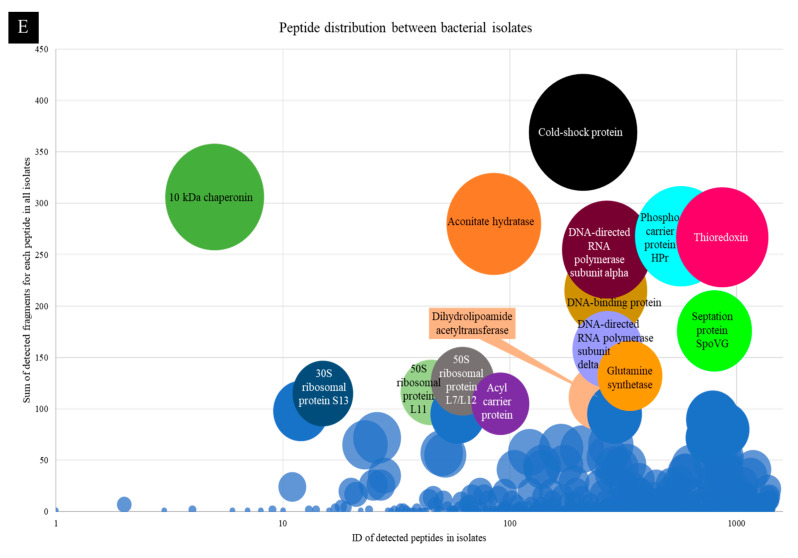
Antimicrobial peptide analysis. Top: Electrophoresis of bacterial supernatants on Tris–Tricine gels. (**A**,**B**) One half of the gel containing bioactive supernatants of the isolates 32YE and 9th, respectively, was challenged with *C. michiganensis* as a “gel plate assay” and the other half of the gel, with identical samples, was stained with Coomassie Brilliant Blue. (**C**,**D**) Gel plate assay of the isolates 33YE or 32LM, respectively, tested against *L. monocytogenes* and its half-stained gel with identical samples to visualize and recover the protein band(s) for further characterization by MS/MS. (**E**) MS/MS data from all peptides and protein fragments’ excised bands from all isolates (Supplementary File S6, Table S1). CS: crude supernatant; OP: organic phase; AP: aqueous phase; L: protein standard.

Overall, 22 concentrated supernatants displayed antimicrobial peptide activity in the previous step (Figure 1 Step 7; Figure S2). To achieve better band separation and excision on the electrophoresis gel, chloroform–methanol extraction was carried out prior to gel electrophoresis, enabling the separate application of aqueous and organic phases onto the gels. Fifteen samples showed activity on peptide gels (Step 8, Figure 2; Supplementary File S1, Figure S3) from 50 µL extracts. These were either the concentrated supernatants from each isolate or the chloroform–methanol-fractionated supernatants. However, the antimicrobial activity from six isolates, 25LGS, 44LGF, 8LM, 30LM, 1M1 and 20M, vanished from their supernatants after chloroform–methanol extraction.

Excised bands from each isolate were further analyzed by tandem mass spectrometry. According to the gel electrophoresis photos and MS/MS data, the size of active AMPs varied between 6 to 13 kDa for different bacterial isolates. Fifteen peptides and fragmented proteins were found to be frequently detected in all bacterial isolates (Figure 2E) and putative AMPs were identified in the following genera.

***Bacillus*** species contained a wide range of potential bioactive peptides, including cold-shock protein, thioredoxin (TRX), DNA-binding protein, phosphocarrier protein HPr, 10 kDa co-chaperone (co-chaperonin GroES), septation protein SpoVG hypothetical proteins, and 30S and 50S ribosomal proteins. ***Lysinibacillus*** species revealed the presence of peptides from antibiotic biosynthesis monooxygenase, septation protein spoVG, thioredoxin, RNA-binding proteins and hypothetical proteins with potential antimicrobial activity. Potential antimicrobial peptides in ***Sporosarcina*** species included thioredoxin, cold-shock proteins, 50S ribosomal RNA, septation protein SpoVG and DNA-binding proteins. ***Brevibacillus*** species had 50S ribosomal protein L7/L12 (12.4 kDa) and cold-shock proteins (7.18 kDa) (Table 2 and Supplementary File S2, Tables S1–S8).

### 2.5. Larger Proteins

In most of bands that were excised from gels and analyzed by MS/MS, matches to larger proteins were detected, which was unexpected, compared with the band sizes (~6–13 kDa) with antimicrobial activities in gels. These could be degradation products or background contamination from abundant proteins, or else the bacteria may purposefully have cleaved larger proteins into smaller fragments to use these as AMPs. Through BlastP-matching with amino acid sequences in NCBI (National Center for Biotechnology Information) and estimating the observed protein fragment sizes (from gel electrophoresis), degraded DNA-directed RNA polymerases (subunit alpha and delta, around 8–10 kDa) were observed in nearly all samples, which indicates the potential of these protein fragments for antimicrobial activity (Supplementary File S3, Table S1).

### 2.6. Identification of Small Antimicrobial Peptides through HPLC Fractionation

To examine the presence of smaller peptides outside the range of Tris–Tricine gels, the 24 isolates that had shown AMC activity were also fractionated using HPLC, based on their hydrophobicity. Each fraction was tested for antimicrobial activity using plate bacterial growth inhibition assays. Fractions that inhibited bacterial growth (Supplementary File S1, Figure S4A) were treated by proteinase K to confirm the presence of peptides (Supplementary File S1, Figure S4B). HPLC fractions that had shown AMP activity were analyzed by MS/MS, and most of the samples showed high numbers of small-sized peptides. Only four samples with AMPs bigger than 8 kDa were observed (Supplementary File S4).

### 2.7. Identification of Antimicrobial Metabolites

Only active HPLC fractions from the previous steps were analyzed by GC/MS. After subtracting control (media) peaks from each bacterial isolate’s fraction (Supplementary File S5, Tables S1 and S2), potential antimicrobial metabolites (AMMs) were identified based on the absence of peaks in the medium-only controls; these are listed in Table 3. Among these metabolites, acetic acid, dimethyl sulfoxide (DMSO), 2,3-butanediol and glycerol were the most abundant. It is noteworthy that these compounds were not detected in the control media, confirming that they originated from the microbes and were not artefacts. Of greater interest were several diketopiperazines (DKPs), especially 3-isobutyl hexahydropyrrolo[1,2-a]pyrazine-1,4-dione and 2-acetyl-3-methyl-octahydropyrrolo[1,2-a]piperazine-1,4-dione, which showed a 4.5–13-fold increase and a 5–12.5-fold increase, respectively, compared with the other samples.

3-Isobutyl hexahydropyrrolo[1,2-a]pyrazine-1,4-dione was detected in media-only control samples (LM and YEP), but with a lower area of total ion current (TIC). 3-Isobutyl hexahydropyrrolo[1,2-a]pyrazine-1,4-dione and 2-acetyl-3-methyl-octahydropyrrolo[1,2-a]piperazine-1,4-dione were detected in *Cmm* culture samples with different retention times, which may indicate different isomer or analogs of these compounds. In addition, *Pst* produced several compounds that were not detected in any fraction (Supplementary File S5, Table S1).

**Figure 3:**
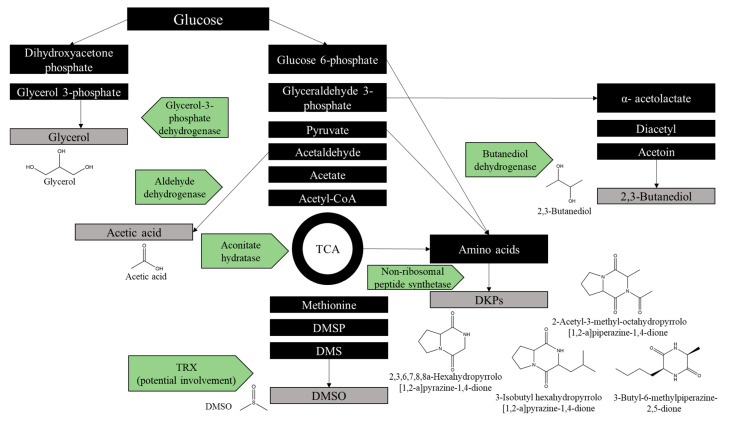
Potential synthetic pathway for AMMs that presented protein fragments that may have originated from metabolic pathway enzymes. The figure shows the proposed AMM pathways that biosynthesize both metabolites (based on GC/MS data) and protein fragments of metabolic enzymes (based on MS/MS data). Pathways include the tricarboxylic acid cycle (TCA), as well as the butanediol, glycerol and acetic acid pathways.

By matching amino acid sequencing data and metabolite profiles, the tricarboxylic acid (TCA), glycerol and butanediol pathway enzymes, including TRXs, aconitate hydratase, dihydrolipoamide acetyltransferase, butanediol dehydrogenase, aldehyde dehydrogenase and glycerol-3-phosphate dehydrogenase, were detected (Figure 3).

## 3. Discussion

We isolated bacteria from biodiverse environmental samples (60 from a soil sample and eight from food spoilage microbial communities) on seven different media to obtain a wide microbial coverage (Figure 1, Step 1). To shorten the process and minimize laborious work, colonies were directly streaked onto a gridded plate to obtain single colonies (Figure 1, Step 2), then pure bacterial cultures were screened against *Pst*, *Cmm*, *L. monocytogenes* and *E. coli* lawn plates to identify zones of inhibition, which would indicate the production of AMCs (Figure 1, Step 3; Supplementary File S1, Figure S1).

Certain bacteria only produce AMCs in the presence of competing species, known as facultative antibiotic production [12,15]. Tyc et al. have also shown, by using an interaction method based on co-culturing two soil isolates using a colony picker robot, that around 42% of soil bacteria inhibited the growth of pathogens on an agar overlay assay [13]. In our study, once we identified potential AMC-producing bacteria by co-culturing them on agar, we then co-cultured living phytopathogens (*Pst* and *Cmm*) as safe (for humans) inducers to produce AMCs on a larger scale in a liquid culture (Figure 1, Steps 4 and 5). Interestingly, more AMCs were produced by stimulation with *Cmm*, possibly due to elicitors present on the cell wall of these Gram-positive bacteria. Similar to the previous study by Tyc et al. [13], we observed higher antimicrobial activity against Gram-positive bacteria than Gram-negative bacteria. We then concentrated a small amount of the culture (16 mL) to ~1–2 mL (Figure 1, Step 6) to identify the components responsible for antimicrobial activity (peptides or metabolites) (Figure 1, Step 7; Supplementary File S1, Figure S2). The broth co-culture method was successful in maintaining the biocontrol activity, as more than 38% (26 out of 68) of the biocontrol isolates selected from the plates also induced AMCs in the liquid culture, although environmental factors such as the pH, the amount of culture and the stimulating pathogens may lead to different AMC profiles in biocontrol bacteria [16].

Peptides from the co-culture supernatant were separated on gel electrophoresis (Figure 1, Step 9; Figure 3 and Supplementary File S1, Figure S3), with the bands exhibiting antimicrobial activity analyzed by MS/MS (Supplementary File S2, Tables S1–S8). Identification of peptides was based on matched amino acid sequence identity and observed size during gel electrophoresis. In our *Bacillus* spp. collection, we found a set of cold-shock proteins. Interestingly, a cold-shock protein was recently characterized as a bacteriocin in *Bacillus thuringiensis* [17]. We also identified TRX, a small redox protein with many important roles in biological processes, including antioxidant functions, reducing reactive oxygen species and bacterial growth [18,19]. Recently, it was discovered that *Arabidopsis* TRX has antifungal activity against pathogenic fungi and yeasts [20]. Other potential AMPs we found included 10 kDa co-chaperones (co-chaperone GroES), which assist with protein folding and functionalizing proteins, such as heat-shock proteins [21]. New evidence has suggested that 10 kDa co-chaperones can increase the tolerance of bacteria to antibiotics, such as aminoglycoside antibiotics; this could be a mechanism of defense to outcompete the pathogens [22]. The phosphocarrier protein HPr may have a pleiotropic role (in this case, bacteria–bacteria interactions), probably through the innate immune response [23]. The septation protein SpoVG is a 10 kDa RNA-binding protein that negatively regulates asymmetric cell division, and it is considered to be a global post-transcriptional regulator that can impact cell growth, virulence and survival [24]. Production of large bacteriocin in *L. mangiferihumi* has been reported previously, and the significant amount of antibiotic biosynthesis monooxygenase in *Lysinibacillus* spp. may indicate the potential role of this enzyme in antibiotic production [25,26]. In our study, *Brevibacillus* was the only genus that produced two potential AMPs with the predicted sizes: cold-shock protein and 50S ribosomal proteins L27 and L30, which have antimicrobial activity, according to previous studies [17,27]. In line with the other new biocontrol bacteria that have been mentioned above, to our knowledge, there is no information about *Sporosarcina* species’ antimicrobial peptide activities that are potentially derived from thioredoxin, cold-shock proteins, 50S ribosomal RNA, septation protein SpoVG and DNA-binding proteins.

Despite the size of the holoenzyme (21.48–34.95 kDA), two cleavage products (protein fragments) from the alpha and delta subunits of the DNA-directed RNA polymerase were observed around 8–10 kDa in most gel electrophoresis bands for 10 out of 16 bacterial supernatants (Figure 3; Supplementary File S3, Table S1). Although there were other peptides present, it is possible that these cleavage products may possess antimicrobial activity, such as lactoferrin, which also presents a protein fragment of a larger protein [28]. Thus, we speculated that these larger proteins in bacteria, when not needed, may degrade to bioactive protein fragments that may possess antimicrobial activity. Further studies are required to determine if these protein fragments are actively secreted by live cells or if proteins released from lysed cells are subsequently degraded. In both cases, the purpose could be to inhibit the growth of other microorganisms. It can even be envisaged that co-cultivation with live pathogens led to the lysis of the producing biocontrol bacteria whose degradation products were antimicrobial.

Acetic acid, DMSO and glycerol are widely known for their bactericidal activities [29,30,31]. 2,3-Butanediol is a volatile compound that has plant growth-promoting properties and indirect antifungal properties [32]. All four metabolites were highly abundant in the GC/MS fractions analyzed in the present study (Supplementary File S5, Table S2). The potential antimicrobial properties of different DKPs have been summarized and reported previously [33,34]. Recently, the antimicrobial activity of 2,3,6,7,8,8a-hexahydropyrrolo[1,2-a]pyrazine-1,4-dione has been confirmed in the sponge-associated bacteria *Bacillus tequilensis* [35]. Several scientific reports supported the idea that bioactive extracts containing 3-isobutyl hexahydropyrrolo[1,2-a]pyrazine-1,4-dione have antifungal and anti-inflammatory effects; here, we strongly suggest its potential involvement in the bacterial defense system as an AMM [36,37]. Furthermore, to our knowledge, the present study, for the first time, suggested the potential contribution of 2-acetyl-3-methyl-octahydropyrrolo[1,2-a]piperazine-1,4-dione to bacterial defense as an AMM. However, through the method of induction used in this study, the production of AMMs by *Cmm* as an inducer cannot be fully excluded as a self-defense mechanism against the biocontrol bacteria. Two potential AMMs were also present in the *Cmm*-only control, albeit at lower concentrations (highlighted in Supplementary File S5, Table S1). Further validation of antimicrobial activity should be performed with synthetic 3-isobutylhexahydropyrrolo[1,2-a]pyrazine-1,4-dione and 2-acetyl-3-methyl-octahydropyrrolo[1,2-a]piperazine-1,4-dione.

Based on data from amino acid sequencing by MS/MS, several proteins such as TRXs, aconitate hydratase, dihydrolipoamide acetyltransferase, butanediol dehydrogenase, aldehyde dehydrogenase and glycerol-3-phosphate dehydrogenase were detected (Figure 2E; Supplementary File S6, Table S2). Glycerol can be synthesized from glucose, and the related enzyme has been detected as glycerol-3-phosphate dehydrogenase in the MS/MS data (Figure 2E). Furthermore, acetic acid- and 2,3-butanediol-synthesizing enzymes, namely aldehyde dehydrogenase and butanediol dehydrogenase, respectively, were found in the MS/MS data (Figure 2E, Supplementary File S6, Table S2). DMSO can be produced by marine bacteria and yeast from methionine [38,39]. Moreover, it has been shown that TRXs have reductase and oxidase activities [40,41]. Although TRXs’ reduction activity in converting dimethyl sulfoxide (DMSO) to dimethyl sulfide (DMS) has been proven, their oxidase activity in DMSO production has not been confirmed yet. DKPs are cyclic peptides that are often biosynthesized by non-ribosomal peptide synthase from two amino acids [42,43]. Thus, potential defense synthetic pathways for the AMMs that have been detected by GC/MS can be suggested and proposed based on the proteins detected in the MS/MS data (Figure 3).

## 4. Materials and Methods

### 4.1. Isolation of Bacteria from Soil and Food Spoilage Material Using Different Media

Clay-rich soil was collected from Tennyson, Queensland, Australia (27°31′37.0″ S 152°59′51.7″ E). Food spoilage samples included mixed vegetable and fruit scraps from a compost bin. Samples were suspended in water and 10× serial dilutions were made five times. Next, 100 µL of the last three dilutions were plated on seven different media, including LM (liquid medium), mannitol salt, MacConkey agar, Thornton’s medium, YEP (yeast extract peptone), MRS (De Man, Rogosa and Sharpe agar) and a new AG medium containing microalgal powder (Supplementary File S1, Table S3). After obtaining colonies in the mannitol salt, MacConkey agar, AG medium and Thornton’s medium plates, isolates were cultivated in LB (Luria-Bertani) broth.

### 4.2. Plate Pathogen Inhibition Assay

In the first step, around 600 single different colonies were transferred with toothpicks to LB, LM or YEP media using a sterile toothpick to obtain pure isolates to ensure they could be used for co-cultivation. In the second step, 2 µL of liquid culture of each isolate was inoculated onto a petri dish with a grid and co-cultured with a bacterial lawn of pathogens (Supplementary File S1, Figure S1). These included food pathogens (incubated at 37 °C), and plant pathogens (incubated at 28 °C).

### 4.3. Identifying Potential Biocontrol Bacteria

The bacterial isolates that showed a zone of inhibition in the plate pathogen inhibition assays were subjected to DNA extraction. PCR products were amplified by Phusion High-Fidelity (New England BioLabs, Ipswich, MA, USA) following the manufacturer’s protocol for 16S rDNA using the universal primers 27F and 1492R [44]. The amplicons were cleaned up using Wizard SV Gel and PCR Clean-Up System (Promega, Madison, WI, USA) according to the manufacturer’s instructions, and DNA-sequenced by Macrogen, (Seoul, Korea).

### 4.4. AMC Induction Assay

Liquid cultures of potential biocontrol bacteria and pathogens were grown in separate Corning tubes or flasks on Day 1 at 28 °C, then one 4 mL portion of the pathogen culture was added to three portions (12 mL) of the biocontrol bacterial culture in a total volume of 16 mL, followed by cultivation for 1 more day at 28 °C. On the second day, bacterial pellets (including mixed pathogens and biocontrols) bacterial biomass were removed by centrifugation. The supernatant was collected, freeze-dried and filter-sterilized to obtain a concentrated and sterile bacterial supernatant of ~1–2 mL (concentrated crude supernatant). To reconstitute the freeze-dried material, 1–2 mL of deionized water was added per sample.

### 4.5. Antibacterial and Peptidase Assays

Concentrated supernatants from the AMC induction assays were subjected to plate pathogen inhibition assays against *L. monocytogenes* (ATCC 7644), *E. coli* (ATCC 25922), *Pst* and *Cmm*. Briefly, pathogens were grown overnight and then spread on the plates’ surface by using cotton swabs to obtain bacterial lawns on a sucrose medium (Supplementary File S1, Table S3). Prior to the assay, in different tubes, 50 µL of the supernatant was treated with proteinase K for 1 h at 37 °C, and the reactions were deactivated by heat at 95 °C for 1 h; the same temperature conditions were applied to the supernatant stocks (without proteinase K treatment). After that, 10 µL of the supernatant and the proteinase K-treated supernatant was spotted on plates with a 10 µL pipette and incubated at 28 °C for plant pathogens and 37 °C for food pathogens. Growth inhibition zones of 10 mm or larger were considered to possess antimicrobial activity.

### 4.6. Peptide Gel Microbial Inhibition Assay

To identify new AMPs from bacteria (bacteriocins), 100 µL of the concentrated crude supernatant was first mixed with 400 µL methanol and then 100 µL chloroform. The mixture was vortexed and 300 µL of deionized water was added to each sample. Samples were centrifuged at maximum speed (20,000× *g*) and the phases (organic and aqueous) were separated in new tubes using a 100 µL pipette. Tubes were dried in a Speed-Vac (Labconco CentriVap, catalog no. 7701020) at 45 °C at 1700 rpm, and then the pellet in each tube was resuspended in a 50 µL PBS (phosphate-buffered saline) solution. We prepared 50 µL of the induced concentrated crude supernatant, 50 µL of the organic phase (methanol–chloroform (1:1) extraction) and 50 µL of the aqueous phase (methanol–chloroform (1:1) extraction). To each sample, 47.5 µL of a Laemli buffer, including a 47.5 µL 2X SDS (sodium dodecyl sulfate) buffer (0.125 M Tris-HCl at pH 6.8, 20% glycerol, 4% SDS and 0.02% bromophenol blue) and 2.5 µL of β-mercaptoethanol, was added and incubated for 5 min at 95 °C. The samples were then loaded onto a 16.5% Mini-PROTEAN Tris–Tricine gel (BioRad, Hercules, CA, USA), which was subjected to gel electrophoresis at 135 V for 45 min.

After electrophoresis, the gel was divided into two halves. One half was stained with Coomassie Brilliant Blue (BioRad, Hercules, CA, USA) for 30 min and destained with distilled water for 3 h under mild agitation. The second half was used for fixation with 20% isopropanol and 10% glacial acetic acid for 2 h in a shaker incubator, which was followed by a 3 h distilled water wash. To perform peptide gel microbial inhibition assays, the second half of the gel was placed on a petri dish. A total of 10 µL of the fully grown *L. monocytogenes* culture was added to 10 mL TSA (trypticase soy agar) (Supplementary File S1, Table S3) and molten agar (0.75%) and poured on the gel in the Petri dish. The same procedure was performed for *Cmm,* except that a sucrose medium (Supplementary File S1, Table S3) was used instead.

### 4.7. Partial Characterization of Antimicrobial Peptides by Tandem Mass Spectrometry

In the first step, to identify antimicrobial peptides, 44 peptide bands were excised from gels. Bands were chosen based on antimicrobial activity in the peptide gel microbial inhibition assays from the stained half, where they were excised from the same region in the gel. After trypsinization of the peptides, they were analyzed by LC-MS on a Shimadzu Nexera uHPLC (Kyoto, Japan) coupled to a Triple Tof 5600 mass spectrometer (ABSCIEX, Concord, ON, Canada) equipped with a duo electrospray ion source. A total of 10 µL of each extract was injected onto a 2.1 mm × 100 mm Zorbax C18 1.8 µm column (Agilent, Santa Clara, CA, USA) at 200 µL/min. Linear gradients of 1–80% Solvent B over 36 min at a 200 µL/minute flow rate, followed by a steeper gradient of 80% to 98% Solvent B in 3 min, were used for peptide elution. Solvent B was held at 98% for 2 min for washing the column and returned to 1% Solvent B for equilibration prior to injection of the next sample. Solvent A consisted of 0.1% formic acid (aq) and Solvent B contained 90/10 acetonitrile/0.1% formic acid (aq). The ion spray voltage was set to 5,500 V, with a declustering potential (DP) of 100 V, a curtain gas flow of 25, nebulizer gas 1 (GS1) 50, GS2 to 60, the interface heater at 150 °C and the turbo heater set to 500 °C. The mass spectrometer acquired 200 ms full-scan TOF-MS (Time of Flight Mass Spectrometry) data, followed by up to 10 200 ms full-scan product ion data in the information-dependent acquisition (IDA) mode. Full-scan TOF-MS data were acquired over the mass range 300–2000; for product ion MS/MS, the range was 100–1800. Ions observed in the TOF-MS scan exceeding a threshold of 100 counts and a charge state of +2 to +5 were set to trigger the acquisition of the product ion MS/MS spectra of the resulting 10 most intense ions. The data were acquired and processed using Analyst TF 1.6 and Protein Pilot v 4.5b software (SCIEX, Framingham, MA, USA).

In the second step, after preparative HPLC fractionation, the fractions were freeze-dried and then tested for antimicrobial activity in plate assays. The fractions whose antimicrobial activity vanished under proteinase K treatment were subjected to further identification by uHPLC, mass spectrometry and protein identification by checking the mass spectrum in advance using the mass spectrometer-acquired 500 ms full-scan TOF-MS data over the mass range 400–2000. The data were acquired and processed using Analyst TF 1.6 (SCIEX, Framingham, MA, USA).

### 4.8. Amino Acid Data Analysis

Amino acids sequences (peptides and protein fragments) from excised bands, processed by Analyst TF 1.6 and Protein Pilot v 4.5b software against each species or genus, were pooled and plotted (Figure 2). For each bacterial genus, the 10–15 highest peptide reads with a confidence level of 99% (based on the Sciex software used) and lower (if part of the same sequence) were BlastP-searched. The best matches that covered almost all the short peptide reads based on expected size in the electrophoresis gel were considered as putative AMPs.

### 4.9. HPLC (RP-HPLC)

HPLC was conducted based on hydrophobicity by Prominence Shimadzu, Japan. Prior to actual HPLC, 400–800 µL of the sample was separated by a Phenomenex Strata C18-E cartridge (100 mg/1 mL) column into 3 different fractions: the loading (1000 µL of the supernatant passed the cartridge), washing (0.05% TFA in water) and eluent (0.05% TFA in acetonitrile) fractions. The antimicrobial activity of these three fractions was tested against *Cmm* in plate bacterial growth inhibition assays. After that, all fractions were lyophilized and resuspended in Milli-Q water, then, the active fractions were further fractionated by HPLC using a C18 column (Vydac 218TP 300 Å, 5 μm, 4.6 mm i.d. × 250 mm; Lot No. E100621-2-1, Serial No. 610120256) at 0.3 mL/min using a gradient of 0–80% B (A, H_2_O 0.05% TFA; B, 90% CH_3_CN 10% H_2_O 0.045% TFA) over 60 min, and the absorbance was measured at 215 nm and 280 nm. The fractionation started during sample loading onto the column and during the gradient from Time 0 to 60 min in 5 min intervals. All fractions from each sample were examined by growth inhibition assays with *Cmm*, and plate inhibition zones of ≥5 mm were considered as validation of the antimicrobial activity.

### 4.10. GC/MS

All fractions whose antimicrobial activity was not affected by proteinase K were subjected to GC/MS armed with a Restek (Bellefonte, PA, USA) Rtx-5MS (Crossbond diphenyl dimethylpolysiloxane) capillary column (30 m × 0.25 mm × 0.25 mm). A total of 1 μL was injected using a split mode, with a split ratio of 10, an injector temperature of 240 °C and an initial oven temperature of 160 °C. Helium was applied as the carrier gas at a constant linear velocity of 46.6 cm/s. The oven temperature program was set to isothermal 160 °C for 1 min, with a temperature gradient of 160–300 °C at 10 °C/min. The mass spectrometer was operated with an ion source temperature of 200 °C and an interface temperature of 300 °C. The analysis was carried out in a full-scan mode with a mass range of 42–500 *m/z*. The peaks were fully or partially characterized by searching the National Institute of Standards and Technology (NIST) 14 library, with similarity indices (SI) > 85.

## 5. Conclusions

We have developed a new pipeline to force biocontrol bacteria to produce new potential bioactive compounds. This was achieved by combining a series of screening and characterization tools, including an AMC induction method in a liquid culture, AMP gel electrophoresis, chemical analysis methods and a bioassay. Future studies should focus on additional validation of the candidate compounds and determine their applicability as useful antimicrobial agents.

## Figures and Tables

**Table 1:** Bacterial isolates from this study with inducible antimicrobial activity.

Isolates Name	Isolates’ LAB ID	*C. michiganensis*	*L. monocytogenes*	*P. syringae*	*E. coli*
*B. pumilus*	18M	AMM	AMM		
	20M	AMP			
	44YE	AMM	AMP		
	8LM	AMP			
	32LM		AMP		
	33LM		AMP		
	35LM		AMP		
*B. subtilis*	1M1	AMP	AMP		
	25LGS	AMP	AMP		
*B. amyloliquefaciens*	33YE	AMM	AMP		
*B. licheniformis*	28M		AMP		
*B. mojavensis*	30LM		AMP		
*B. circulans*	9th		AMP		
*B. methylotrophicus*	46YE	AMP			
	45YE		AMP		
	42LGS	AMM	AMM		
*B. safensis*	35YE	AMM	AMP		
	10th	AMP			
*B. laterosporus*	4YE	AMM	AMP		
*P. peoriae*/*polymyxa*	14th			AMM	AMM
*L. fusiformis*	34LM		AMM		
*L. mangiferihumi*	37LM	AMP	AMM		
*K. pneumoniae*	44LGF	AMP			
*S. aquimarina*	32YE	AMP	AMP		
	19YE	AMP	AMP		
*S. saromensis*	39YE	AMP	AMP		
**After adjustment to pH 7**					
*S. saprophyticus*/*xylosus*	36M	AMP		AMM	
	41M	AMP		AMM	
*S. sciuri*	42M	AMP		AMM	
*P. vulgaris*	30MC			AMP/AMM	
*E. ludwigii*	46M	AMM			
*E. cloacae*	42MC			AMP/AMM	
*C. jiangduensis*	28MC			AMP/AMM	
*C. flavescens*	43M	AMM		AMM	
*O. grignonense*/*pseudogrignonense*	34MC			AMP	
	37MC			AMP	

**Table 2:** Putative promising AMPs were identified by MS/MS for different genera (Supplementary File S2, Tables S1–S8).

Genera	Cold-Shock Protein	Thioredoxin (TRX)	DNA-Binding Protein	RNA-Binding Protein	Phosphocarrier Protein HPr	10 kDa Co-Chaperone (Co-Chaperonin GroES)	Septation Protein SpoVG	30S Ribosomal Proteins	50S Ribosomal Proteins	Hypothetical Proteins	Antibiotic Biosynthesis monooxygenase
*Bacillus* spp. (9th, 10th, 32LM, 33LM, 35LM, 33YE, 35YE, 44 YE, 45YE, 46YE)	✓	✓	✓	-	✓	✓	✓	✓	✓	✓	-
*Lysinibacillus* spp. (37LM)	-	✓	-	✓	-	-	✓	-	-	✓	✓
*Sporosarcina* spp. (32YE and 39YE)	✓	✓	✓	-	-	-	✓	-	✓	-	-
*Brevibacillus* spp. (4YE)	✓	-	-	-	-	-	-	-	✓	-	-

**Table 3:** Summary of metabolites from biocontrol bacteria detected by GC/MS with potential antimicrobial activity.

Name of Isolates	Potential AMMs
*Bacillus* spp.	2,3-Butanediol
30LM, 32LM, 35LM	Acetic acid
35YE, 45YE, 46YE,	DMSO
18M, 20M, 10th	Glycerol
	3-Isobutyl hexahydropyrrolo[1,2-a]pyrazine-1,4-dione *^,^ **
	Dihydro-1H-pyrrolizine-3,5(2H,6H)-dione
	(2S)-1-(2-Aminoacetyl)pyrrolidine-2-carboxylic acid
	2,3,6,7,8,8a-Hexahydropyrrolo[1,2-a]pyrazine-1,4-dione
	2-Acetyl-3-methyl-octahydropyrrolo[1,2-a]piperazine-1,4-dione
	1,4,5-Triethyltetrazaborole
	3-Butyl-6-methylpiperazine-2,5-dione
	5-Methylimidazolidine-2,4-dione
*Lysinibacillus* spp.	2,3-Butanediol
37LM, 34LM	DMSO
	Glycerol
	3-Isobutyl hexahydropyrrolo[1,2-a]pyrazine-1,4-dione
	2-Acetyl-3-methyl-octahydropyrrolo[1,2-a]piperazine-1,4-dione
	2,3,6,7,8,8a-Hexahydropyrrolo[1,2-a]pyrazine-1,4-dione
*Paenibacillaceae* spp.	Acetic acid
4YE, 14th	DMSO
	2,3,6,7,8,8a-Hexahydropyrrolo[1,2-a]pyrazine-1,4-dione
	3-Isobutylhexahydropyrrolo[1,2-a]pyrazine-1,4-dione
	3-Butyl-6-methylpiperazine-2,5-dione
*Klebsiella* spp.	DMSO
44LGF	Dimethyl palmitamine

* Also detected in the medium; ** also detected in *Cmm* culture.

## Data Availability

Data is contained within the article or Supplementary Material.

## Supplementary Materials

The following supplemental information is available online https://www.mdpi.com/article/10.3390/ph14121232/s1.

## Abbreviations

AMPs, antimicrobial peptides; AMMs, antimicrobial metabolites, uHPLC, ultra-high-performance liquid chromatography; GC/MS, gas chromatography/mass spectrometry; MS/MS, tandem mass spectrometry; DMSO, dimethyl sulphoxide; DMS, dimethyl sulfide, TRX, thioredoxin; DKPs, diketopiperazines; *Pst*, *Pseudomonas syringae*; *Cmm*, *Clavibacter michiganensis*.
