# Supplementary material for: Biostimulation of Bacteria in Liquid Culture for Identification of New Antimicrobial Compounds
Source: Pharmaceuticals (Basel). 2021 Nov 27;14(12):1232. doi: 10.3390/ph14121232 (PMC8706287; doi:10.3390/ph14121232)
Supplement: Supplementary file 1 [file pharmaceuticals-14-01232-s001.zip › Supplementary 1 - revised.pdf]

Supplementary information:

Biostimulation of bacteria in liquid culture for identification of new antimicrobial compounds

Hooman Mirzaee[1*], Emily Ariens[1], Mark A. T. Blaskovich[2], Richard J Clark[3], Peer M Schenk[1*]

[1]Plant-Microbe Interactions Laboratory, School of Agriculture and Food Sciences, [2]Centre for Superbug Solutions, Institute for Molecular Bioscience, [3]Peptide Chemical Biology Laboratory, School of Biomedical Sciences, The University of Queensland, Brisbane, Queensland 4072, Australia

*Corresponding author email: h.mirzaee@uq.edu.au; p.schenk@uq.edu.au

Table S1 Antagonistic microbial isolates and their lab ID with antimicrobial activity....................2

Table S2 Identification of microbial isolates with antimicrobial activity……….........................3

Table S3 Media used in this study ……..4

Figure S1 Screening and testing of soil and food spoilage microbial isolates …..............................5

Figure S2 Supernatant antimicrobial plate assays………...6

Figure S3 Electrophoresis of bacterial extracts with antimicrobial peptides..................................7

Figure S4 HPLC fractions inhibition assay against *Cmm*. ………...9

Table S1. Antagonistic microbial isolates with antimicrobial activity.

P. syringae	*C. michiganensis*	*L. monocytogenes*	*E. coli*
1YE, 33YE, 41YE, 45YE, 46YE	5LM, 10LM, 11LM, 12LM, 28LM, 30LM, 37LM	8LM, 7LM, 9LM, 27LM, 32LM, 33LM, 35LM	25LGS
28MC, 30MC, 34MC, 37MC, 46MC	15MC, 19MC, 21MC, 22MC, 23MC, 24MC, 38MC, 42MC, 47MC	19YE, 35YE, 39YE, 32YE, 44YE	
14LGF, 12LGF	28M, 36M, 41M, 37M, 42M, 43M, 46M, 47M	1M, 1M1, 18M, 20M, 27M	
34LM	6th, 9th, 10th, 18th	6th	
14th	4YE, 34YE, 46YE		
44LGF	42LGS, 44LGS, 52LGS		
	23MR, 25MR		

Table S2. Identification of microbial isolates with antimicrobial activity.

Isolate ID	Isolate scientific name	Coverage	Identity	Coverage	Identity
1M	*Bacillus subtilis*	98%	98%		
1M1	*Bacillus subtilis/ Bacillus velezensis*	98%	97%	/ 98%	97%
18M	*Bacillus pumilus*	99%	98%		
20M	*Bacillus pumilus*	96%	99%		
27M	*Bacillus safensis*	97%	99%		
28M	*Bacillus licheniformis*	98%	98%		
36M	*Staphylococcus saprophyticus/ Staphylococcus xylosus*	97%	89%	/ 97%	89%
37M	*Staphylococcus sciuri*	98%	99%		
41M	*Staphylococcus saprophyticus*	93%	99%		
42M	*Staphylococcus sciuri*	97%	99%		
43M	*Corynebacterium flavescens*	95%	99%		
46M	*Enterobacter ludwigii*	98%	99%		
47M	*Staphylococcus sciuri*	98%	99%		
15MC	*Pseudochrobactrum kiredjianiae*	98%	98%		
19MC	*Enterobacter ludwigii*	99%	99%		
21MC	*Enterobacter ludwigii*	95%	97%		
22MC	*Enterobacter ludwigii*	92%	98%		
23MC	*Enterobacter cloacae*	99%	99%		
24MC	*Enterobacter ludwigii*	98%	99%		
28MC	*Comamonas jiangduensis*	92%	99%		
30MC	*Proteus vulgaris*	93%	97%		
34MC	*Ochrobactrum grignonense*	91%	97%		
37MC	*Ochrobactrum grignonense/ Ochrobactrum pseudogrignonense*	95%	99%	/ 96%	97%
38MC	*Enterobacter ludwigii*	96%	94%		
42MC	*Enterobacter cloacae*	99%	99%		
46MC	*Enterobacter ludwigii*	99%	99%		
47MC	*Enterobacter ludwigii*	95%	89%		
23MR	*Lactobacillus plantarum*	95%	98%		
25MR	*Lactobacillus hilgardii*	92%	97%		
1YE	*Lysinibacillus fusiformis*	99%	99%		
4YE	*Brevibacillus laterosporus*	94%	99%		
19YE	*Sporosarcina aquimarina*	98%	99%		
32YE	*Sporosarcina aquimarina*	95%	99%		
33YE	*Bacillus amyloliquefaciens/ Bacillus subtilis*	99%	99%	/ 99%	99%
34YE	*Sporosarcina aquimarina*	97%	99%		
35YE	*Bacillus safensis*	95%	99%		
39YE	*Sporosarcina saromensis*	97%	98%		
41YE	*Bacillus amyloliquefaciens/ Bacillus subtilis*	99%	99%	/ 99%	100%
44YE	*Bacillus pumilus*	92%	99%		
45YE	*Bacillus methylotrophicus*	99%	99%		
46YE	*Bacillus methylotrophicus*	97%	99%		
6th	*Bacillus pumilus*	98%	96%		
9th	*Bacillus circulans*	95%	99%		
10th	*Bacillus safensis*	95%	99%		
14th	*Paenibacillus peoriae/ Paenibacillus polymyxa*	98%	97%		
18th	*Bacillus megaterium*	98%	99%		
5LM	*Bacillus pumilus*	96%	97%		
7LM	*Bacillus pumilus*	98%	99%		
8LM	*Bacillus pumilus*	97%	99%		
9LM	*Bacillus safensis*	95%	98%		
10LM	*Bacillus pumilus*	97%	97%		
11LM	*Microbacterium oxydan*	97%	97%		
12LM	*Bacillus pumilus*	98%	99%		
27LM	*Bacillus pumilus*	98%	99%		
28LM	*Bacillus pumilus*	98%	99%		
30LM	*Bacillus mojavensis*	97%	99%		
32LM	*Bacillus pumilus*	95%	100%		
33LM	*Bacillus pumilus*	95%	100%		
34LM	*Lysinibacillus fusiformis*	97%	99%		
35LM	*Bacillus pumilus*	96%	99%		
37LM	*Lysinibacillus mangiferihumi*	98%	99%		
12LGF	*Pseudochrobactrum kiredjianiae*	98%	97%		
14LGF	*Pseudochrobactrum kiredjianiae*	93%	99%		
44LGF	*Klebsiella pneumoniae*	84%	89%		
25LGS	*Bacillus subtilis*	96%	96%		
42LGS	*Bacillus methylotrophicus*	97%	99%		
44LGS	*Bacillus megaterium*	97%	99%		
52LGS	*Bacillus megaterium*	98%	99%		

Table S3. Media used in this study (shown as per L)

LM	Mannitol	MacConkey	Thornton	YEP
Bactotryptone: 10.0 g<br>Bacto yeast extract: 6.0 g<br>K_2HPO_4: 1.5 g<br>NaCl: 0.6 g<br>$MgSO_4.7H_2O$: 0.4 g	NaCl: 75.0 g<br>Proteose Peptone: 10.0 g<br>Mannitol: 10.0 g<br>Beef Extract: 1.0 g<br>Phenol Red: 0.025g<br>Agar: 18.0 g	Peptone: 17.0 g<br>Lactose: 10.0 g<br>NaCl: 5.0 g<br>Proteose Peptone: 3.0 g<br>Bile Salts: 1.5 g<br>Neutral Red: 30.0 mg<br>Crystal Violet: 1.0 mg<br>Agar: 18.0 g	Maninitol: 1.0 g<br>Asparagine: 0.5 g<br>K2Hpo4: 1.0 g<br>KNO3: 0.5g<br>$MgSO_4.7H_2O$: 0.2 g<br>CaCl2: 0.1 g<br>NaCl: 0.1 g<br>$FeCl_3$: 0.002 g<br>Agar: 18.0 g	Bactotryptone: 10.0 g<br>Bacto yeast extract: 10.0 g<br>NaCl: 5.0 g<br>Agar: 18.0 g
**AG***	**MRS**	**TSA**	**Sucrose media**	**LB**
Bactotryptone: 5.0 g<br>Bacto yeast extract: 5.0 g<br>Spirulina Algae Powder: 5.0 g<br>NaCl: 5.0 g<br>Agar: 18.0 g	Dextrose: 20.0 g<br>Peptic Digest of Animal Tissue: 10.0 g<br>Beef Extract: 10.0 g<br>Yeast Extract: 5.0 g<br>Sodium Acetate: 5.0 g<br>Disodium Phosphat: 2.0 g<br>Ammonium Citrate: 2.0 g<br>Tween ® 80: 1.0 g<br>Magnesium Sulfate: 0.1 g<br>Manganese sulfate: 0.05 g	Tryptone: 15.0 g<br>Soytone: 5.0 g<br>NaCl: 5.0 g<br>Agar: 18 g	Sucrose: 20.0 g<br>Peptone: 5.0 g<br>K_2HPO_4: 0.5 g<br>$MgSO_4.7H_2O$: 0.25 g<br>Agar: 18.0 g	Bactotryptone: 10.0 g<br>Bacto yeast extract: 5.0 g<br>NaCl: 10.0 g

* New medium formulation

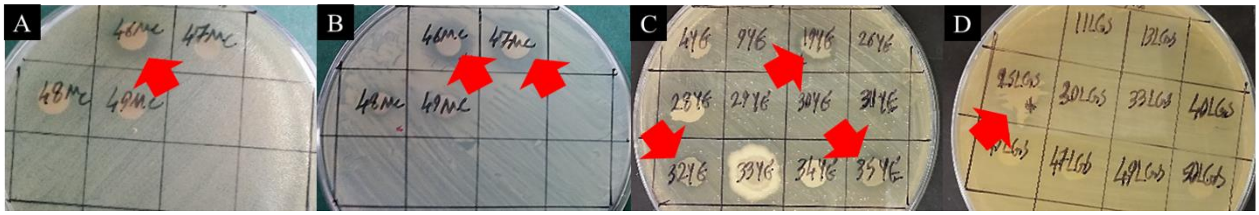

Figure S1. Screening and testing of soil and food spoilage microbial isolates (~600) against (A) *Pst*, (B) *Cmm*, (C) *L. monocytogenes*, and (D) *E. coli*.

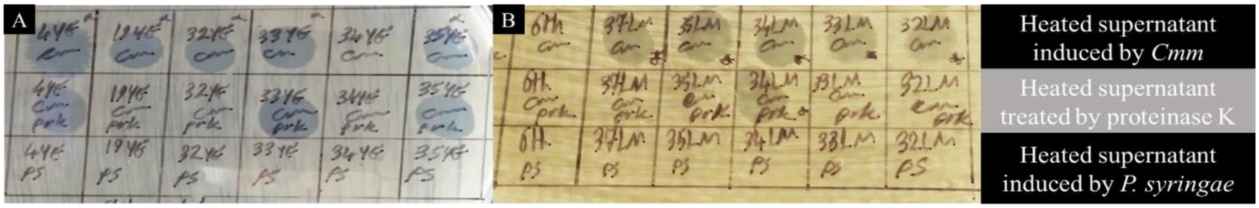

Figure S2. Supernatant antimicrobial plate assays. (A) *Cmm* and (B) *L. monocytogenes* bacterial lawns. The first row shows the isolates' heated supernatant induced by *Cmm*, the second row shows the proteinase K-treated samples, and the third row is the isolates' heated supernatant induced by *Pst.* No growth inhibition was found when the medium only was used.

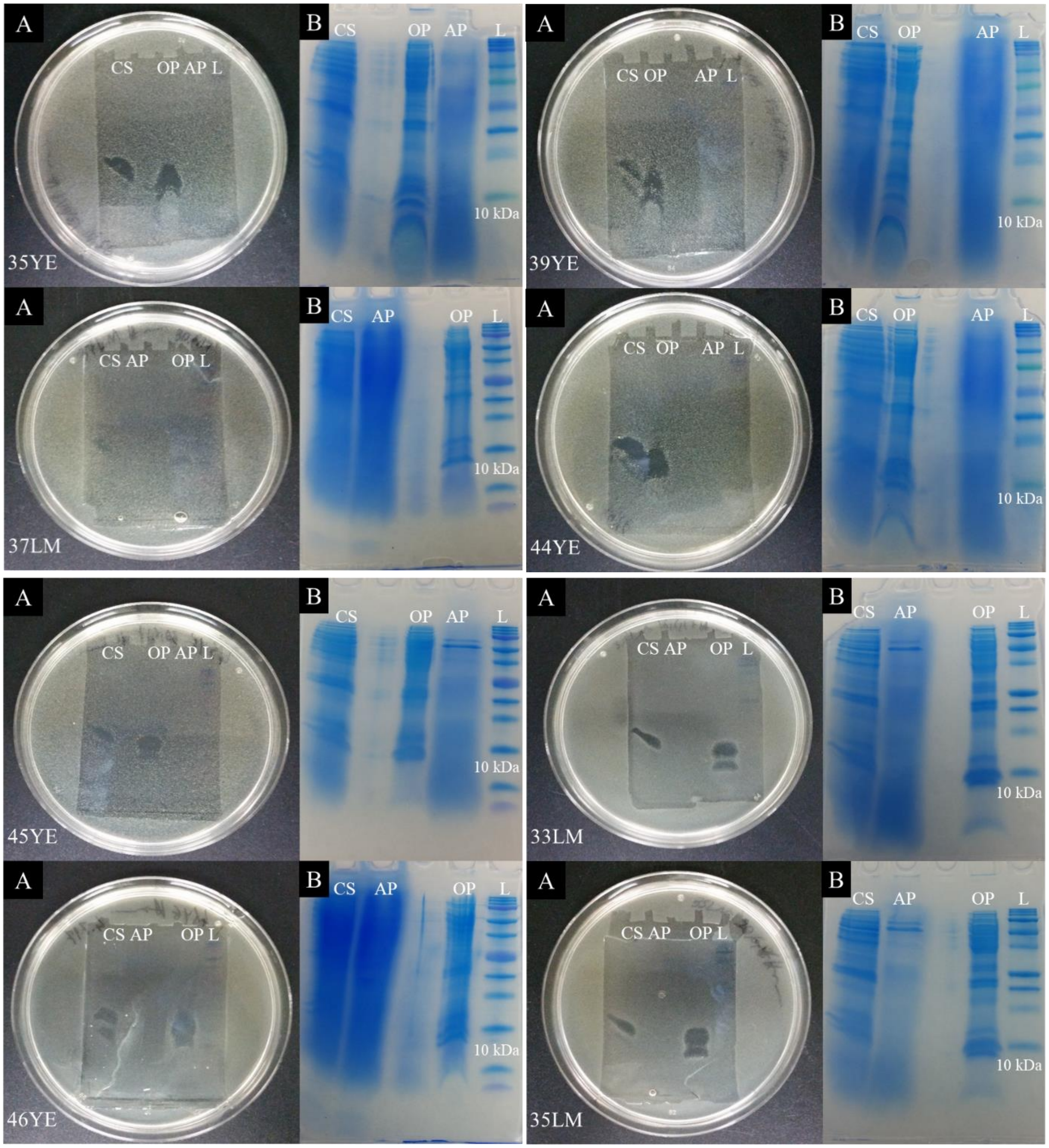
A
CS OP AP L
35YE
B
CS OP AP L
10 kDa
A
CS OP AP L
39YE
B
CS OP AP L
10 kDa
A
CS AP OP L
37LM
B
CS AP OP L
10 kDa
A
CS OP AP L
44YE
B
CS OP AP L
10 kDa
A
CS OP AP L
45YE
B
CS OP AP L
10 kDa
A
CS AP OP L
33LM
B
CS AP OP L
10 kDa
A
CS AP OP L
46YE
B
CS AP OP L
10 kDa
A
CS AP OP L
35LM
B
CS AP OP L
10 kDa

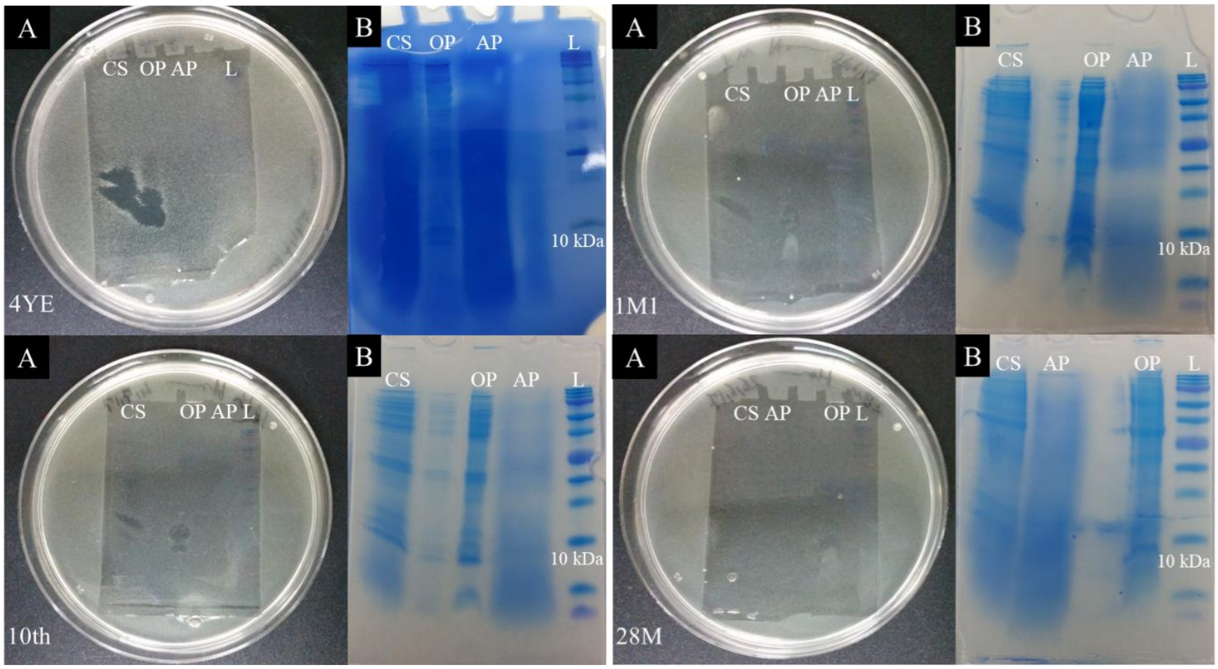

Figure S3. Electrophoresis of bacterial supernatants with antimicrobial peptides. The gel was divided into two halves: (A) One of the halves containing supernatants of different bacterial isolates was challenged with *L. monocytogenes* as a "gel plate assay". (B) The other half of the gel with identical samples was stained with Coomassie Brilliant Blue for visualizing and recovering the band(s) for further characterization. CS: crude supernatant, OP: organic phase, AP: aqueous phase, and L: protein standard.

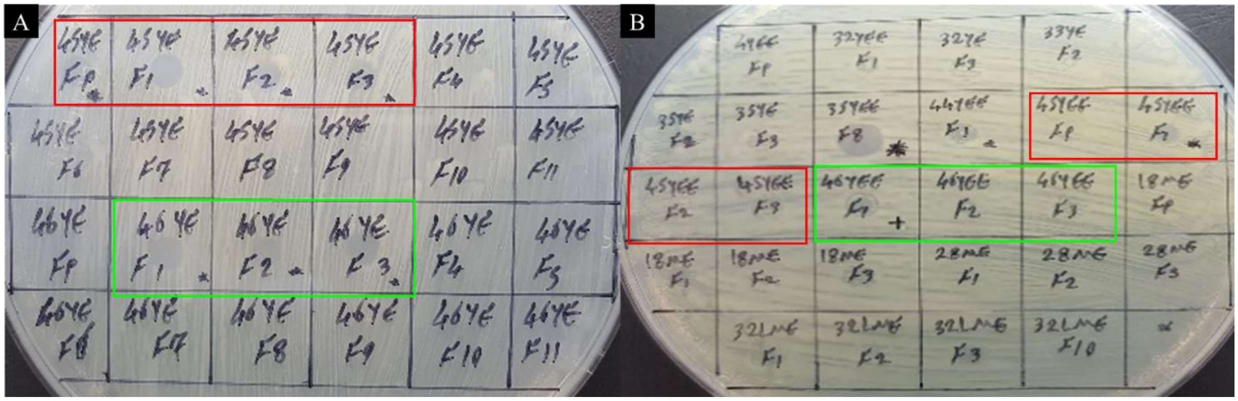

Figure S4. HPLC fractions inhibition assay against *Cmm*. (A) without proteinase K treatment; (B) after proteinase K treatment.
