# Supplementary material for: Biostimulation of Bacteria in Liquid Culture for Identification of New Antimicrobial Compounds
Source: Pharmaceuticals (Basel). 2021 Nov 27;14(12):1232. doi: 10.3390/ph14121232 (PMC8706287; doi:10.3390/ph14121232)

Graph 1. Elution from 4YE (*Brevibacillus laterosporus*)

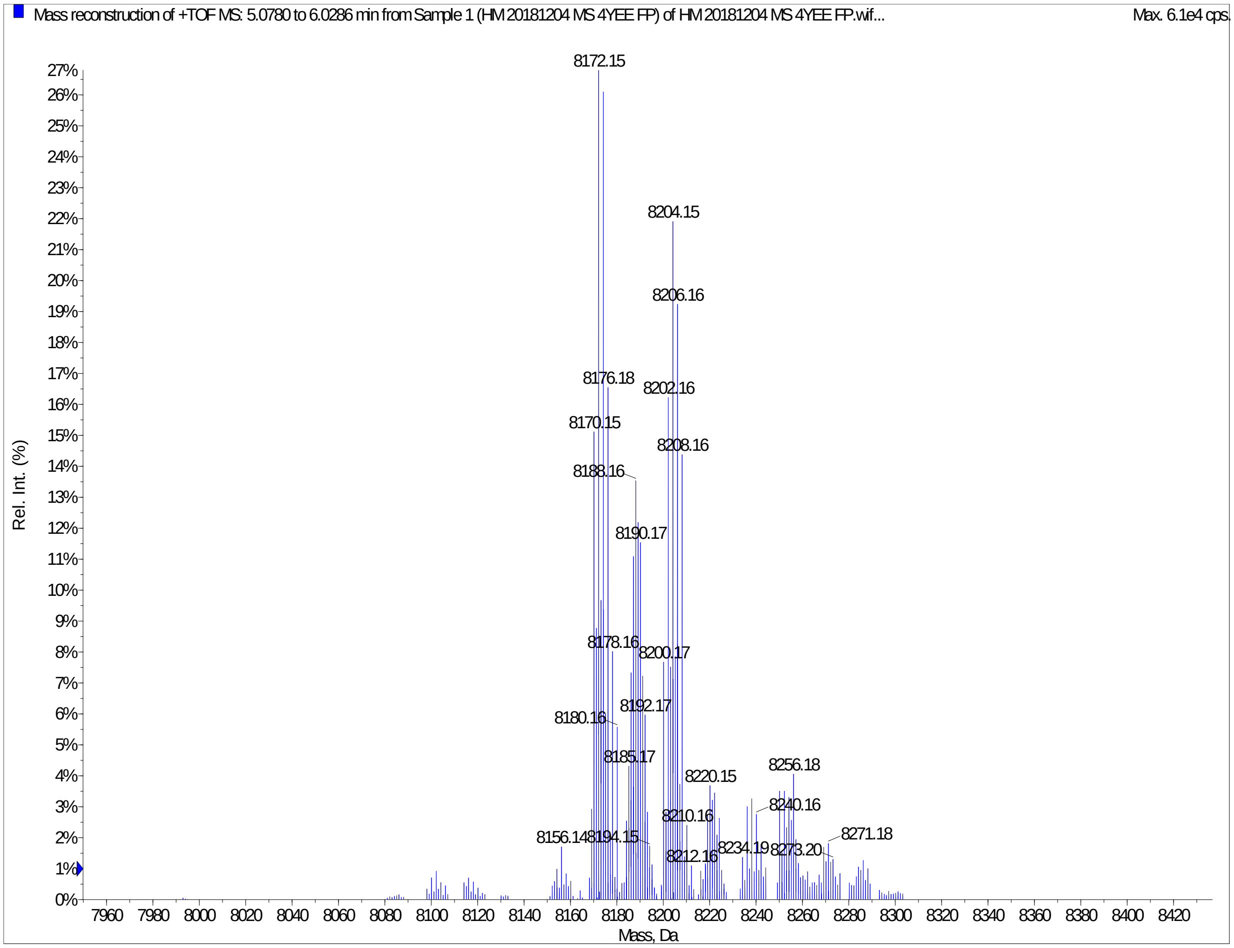

Graph 2. Elution from 35YE (*Bacillus safensis*)

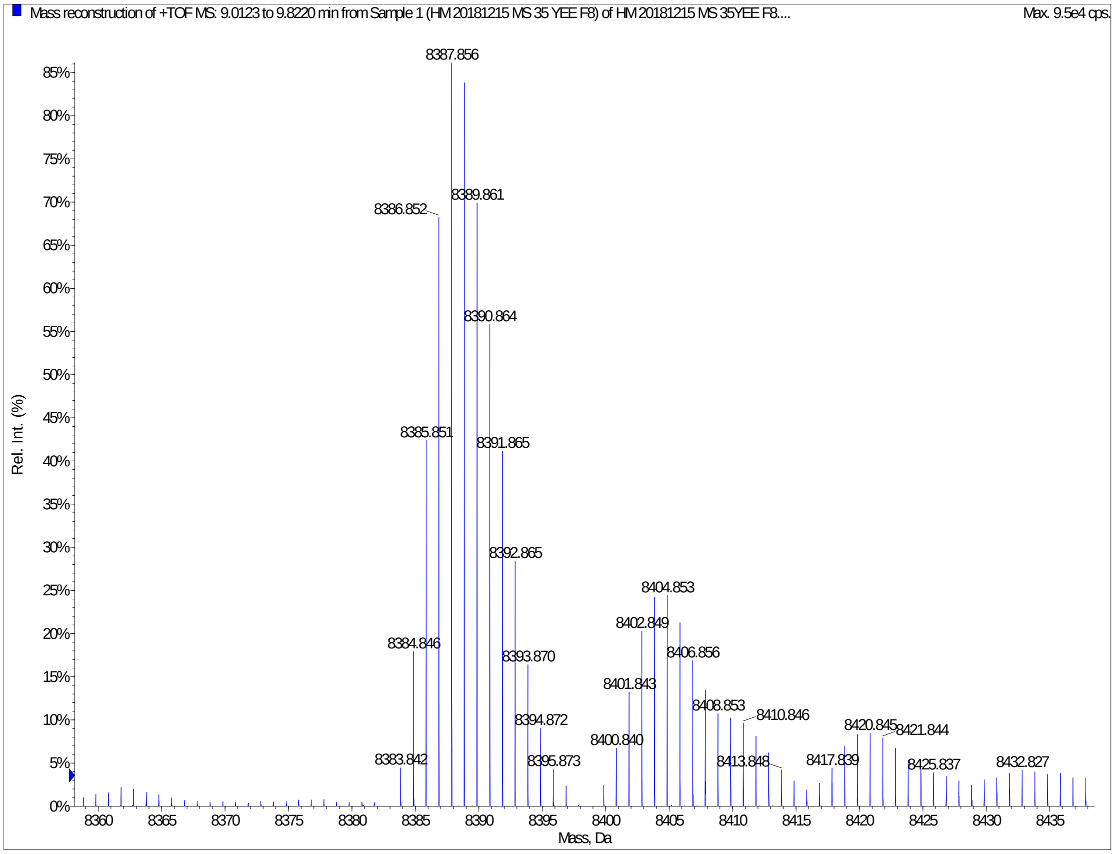

Graph 3. Elution from 10th (*Bacillus safensis*)

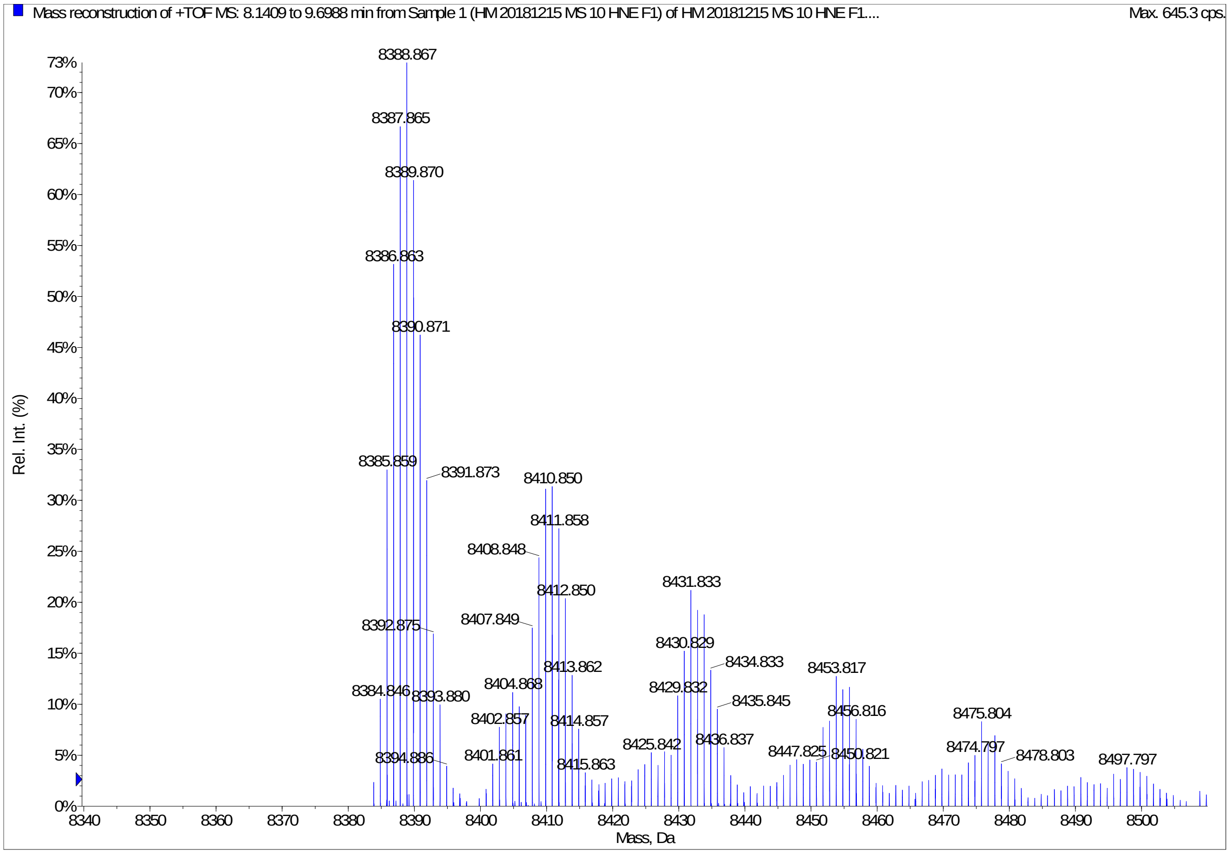

Graph 4. Elution from 35LM (*Bacillus pumilus*)

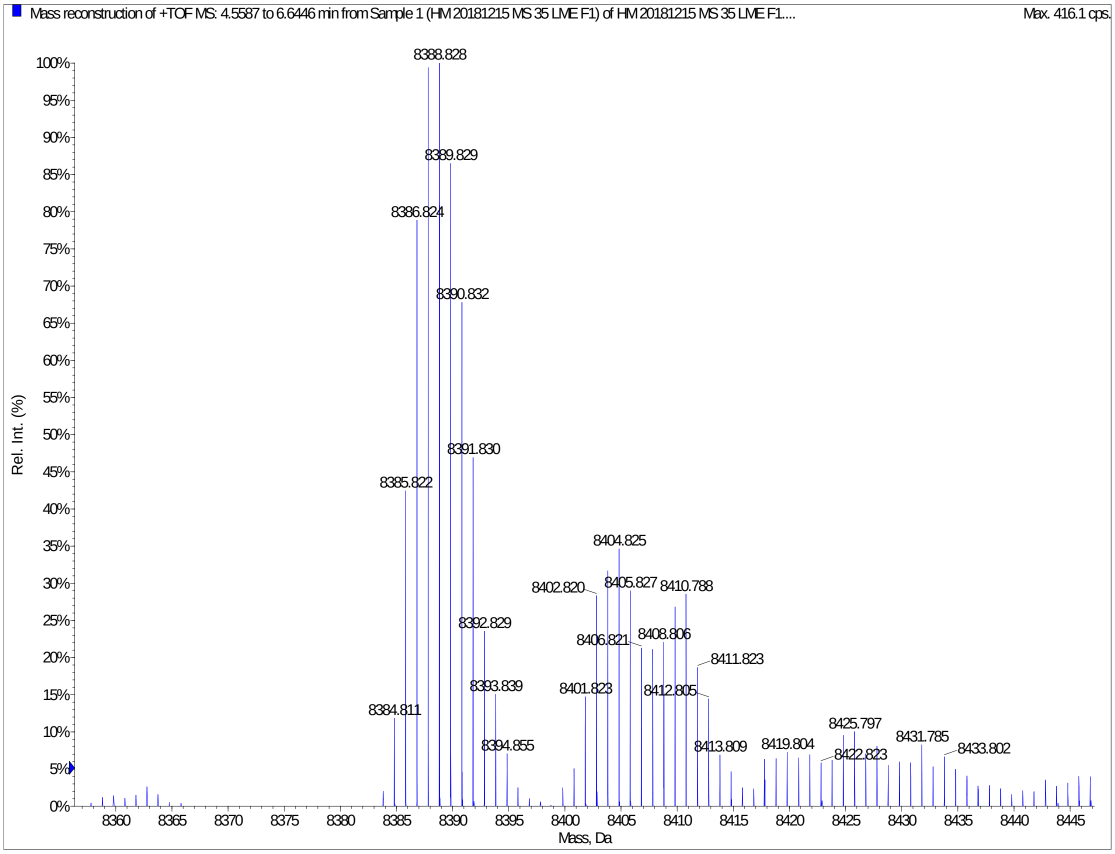

Graph 5. Elution from 35LM (*Bacillus pumilus*)

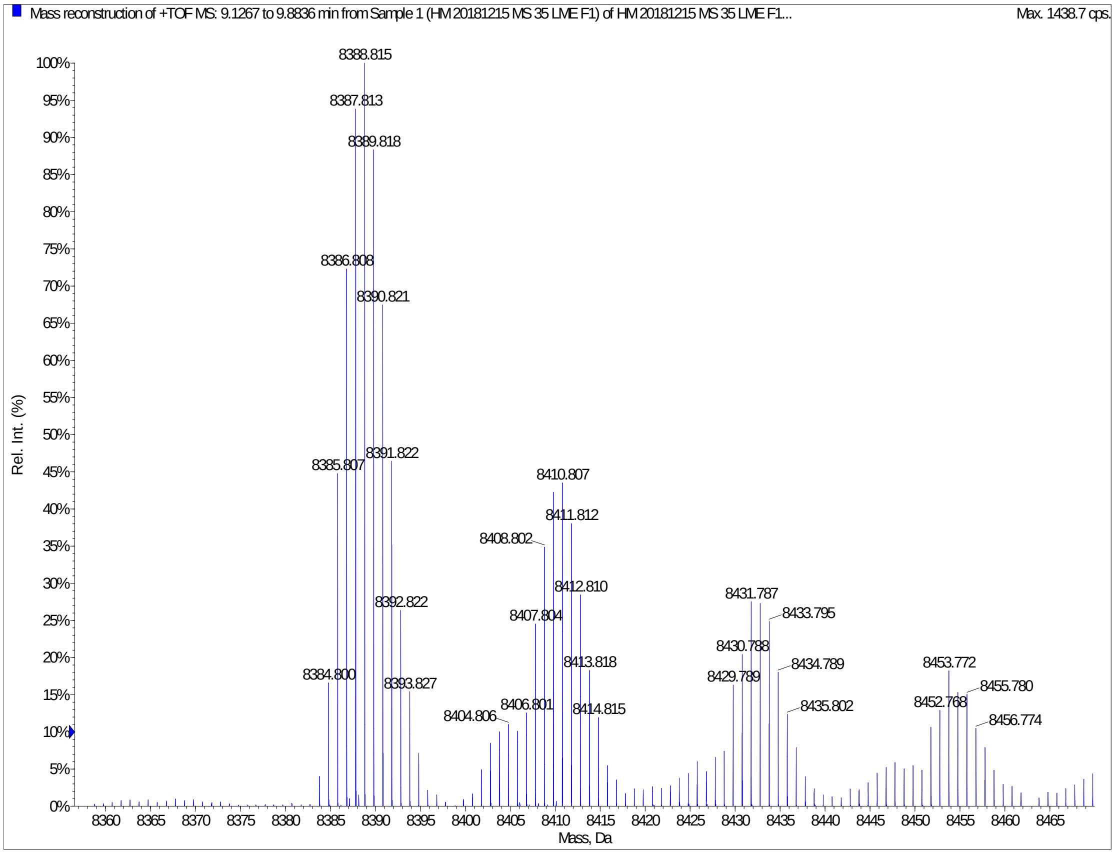

Graph 6. Elution from 35LM (*Bacillus pumilus*)

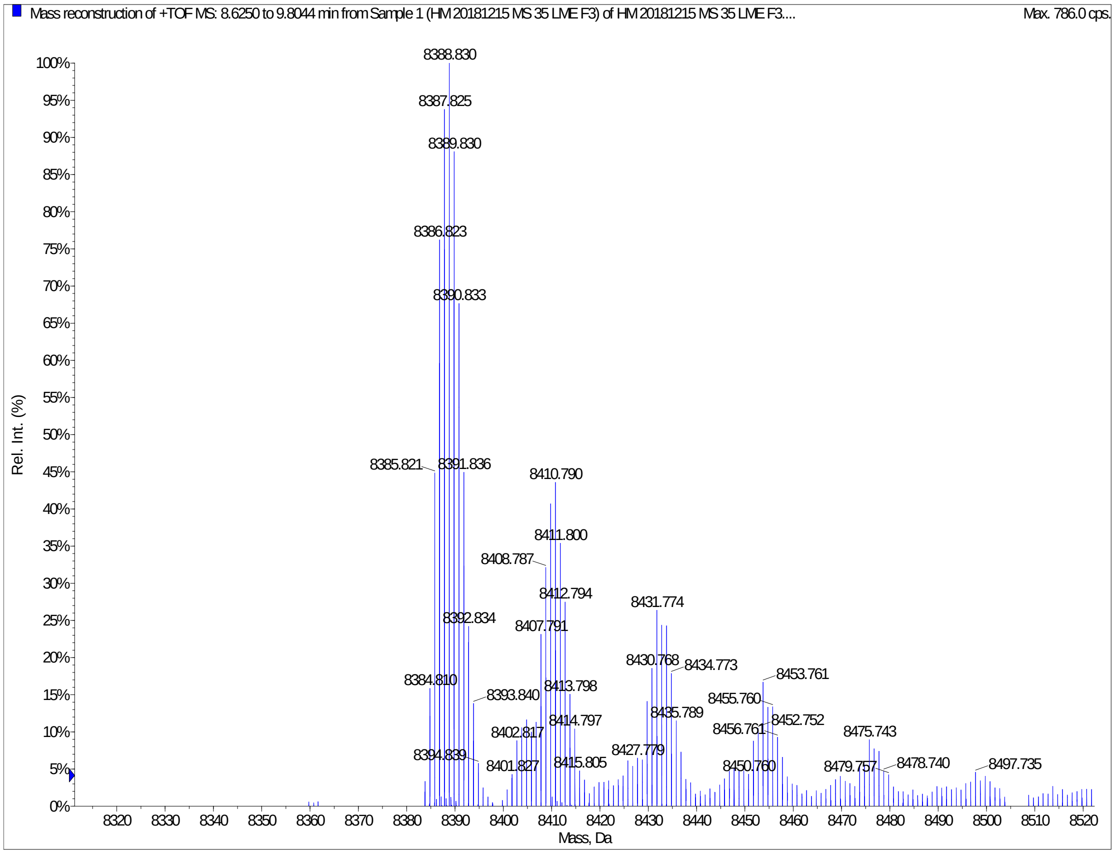

Supplement: Supplementary file 1 [file pharmaceuticals-14-01232-s001.zip › Supplementary 4.pdf]
